# Effectiveness of interventions to increase uptake and completion of treatment for diabetic retinopathy in low- and middle-income countries: a rapid review protocol

**DOI:** 10.1186/s13643-020-01562-9

**Published:** 2021-01-14

**Authors:** Covadonga Bascaran, Nyawira Mwangi, Fabrizio D’Esposito, Charles Cleland, Iris Gordon, Juan Alberto Lopez Ulloa, Ranad Maswadi, Shaffi Mdala, Jacqueline Ramke, Jennifer R. Evans, Matthew Burton

**Affiliations:** 1 London School of Hygiene & Tropical Medicine, Keppel Street, London, WC1E 7HT UK; 2 Kenya Medical Training College, Nairobi, Kenya; 3 The Fred Hollows Foundation, Melbourne, Australia; 4 Centro Mexicano de Salud Visual Preventiva, Mexico DF, Mexico; 5 St Thomas’s Hospital, Westminster Bridge Road, London, SE1 7EH UK; 6 Queen Elizabeth Central Hospital, P.O.Box 95, Blantyre, Malawi; 7 School of Optometry and Vision Science, University of Auckland, Auckland, New Zealand; 8 Moorfields Eye Hospital, London, UK

**Keywords:** Low- and middle-income countries, Diabetic retinopathy, Patient acceptance of health care, Treatment outcome

## Abstract

**Background:**

Vision loss due to diabetic retinopathy can largely be prevented or delayed through treatment. Patients with vision-threatening diabetic retinopathy are typically offered laser or intravitreal injections which often require more than one treatment cycle. However, treatment is not always initiated, or it is not completed, resulting in poor visual outcomes. Interventions aimed at improving the uptake or completion of treatment for diabetic retinopathy can potentially help prevent or delay visual loss in people with diabetes.

**Methods:**

We will search MEDLINE, Embase, Global Health and Cochrane Register of Studies for studies reporting interventions to improve the uptake of treatment for diabetic retinopathy (DR) and/or diabetic macular oedema (DMO), compared with usual care, in adults with diabetes. The review will include studies published in the last 20 years in the English language. We will include any study design that measured any of the following outcomes in relation to treatment uptake and completion for DR and/or DMO: (1) proportion of patients initiating treatment for DR and/or DMO among those to whom it is recommended, (2) proportion of patients completing treatment for DR and/or DMO among those to whom it is recommended, (3) proportion of patients completing treatment for DR and/or DMO among those initiating treatment and (4) number and proportion of DR and/or DMO rounds of treatment completed per patient, as dictated by the treatment protocol. For included studies, we will also report any measures of cost-effectiveness when available. Two reviewers will screen search results independently. Risk of bias assessment will be done by two reviewers, and data extraction will be done by one reviewer with verification of 10% of the papers by a second reviewer. The results will be synthesised narratively.

**Discussion:**

This rapid review aims to identify and synthesise the peer-reviewed literature on the effectiveness of interventions to increase uptake and completion of treatment for DR and/or DMO in LMICs. The rapid review methodology was chosen in order to rapidly synthesise the available evidence to support programme implementers and policy-makers in designing evidence-based health programmes and public health policy and inform the allocation of resources.

**Systematic review registration:**

OSF osf.io/h5wgr

**Supplementary Information:**

The online version contains supplementary material available at 10.1186/s13643-020-01562-9.

## Background

Vision loss due to diabetic retinopathy (DR) can largely be prevented or delayed through treatment. DR programmes focus on regular screening of people living with diabetes and referral to eye care services for further assessment. Patients with vision-threatening diabetic retinopathy (VTDR), including proliferative diabetic retinopathy (PDR) and/or diabetic macular oedema (DMO), are then offered laser treatment and/or intravitreal injections which often require more than one treatment visit [1, 2]. However, the effectiveness of DR treatment is dependent on the timely initiation and completion of the prescribed treatment course.

Different factors—both from the demand and the supply side—can affect treatment uptake and completion [3]. Health systems in low- and middle-income country (LMIC) settings experience limitations in the delivery of high-quality and timely DR treatment, including low numbers of ophthalmologists trained in DR management, inadequate referral systems, limited access to technology and equipment and the lack of health information systems and policies that address DR management [4, 5]. LMIC populations have competing health needs that determine their priorities and health-seeking behaviours in relation to eye health. Barriers like fear, price of services, waiting times or distance can lead to patients missing treatment, or initiating treatment but not completing it, resulting in poor visual outcomes. Low uptake of DR treatment compounds the challenge to prevent visual loss in people with diabetes and wastes resources in already limited health systems [6]. Interventions aiming to improve uptake and completion of DR treatment need to target the relevant factors in the healthcare environment in which the patients are to receive treatment [6].

Access to DR services is a widespread problem in LMICs. Studies of DR services in Pakistan, Oman and China reported that 29.5%, 22.8% and 27.9% of patients respectively did not initiate treatment [7–9]. In the study in China, an additional 17.6% did not complete the treatment course, for reasons including being unaware of the importance of treatment and the need to complete the full course, and fear of treatment [9]. In high-income countries, the proportion of patients not completing treatment tends to be lower than in low-income countries but still significant. A review in the USA reported that approximately 15% of patients did not initiate treatment and 15% of those did not complete it [10].

Laser photocoagulation is a highly effective treatment for PDR and DMO [11–13]. DMO and PDR can be treated with anti-vascular endothelial growth factor (VEGF) via intravitreal injections [14, 15]. Intravitreal steroids, while not indicated as first-line therapy for most eyes with DMO, have been used by some clinicians for eyes that do not adequately respond to anti-VEGF therapy [16, 17]. Anti-VEGF is also indicated in age-related macular degeneration (ARMD) [18]. However, there is evidence that the uptake of anti-VEGF by patients with DMO is significantly lower compared to patients with ARMD [19–21]. People with diabetes often have other co-morbidities that increase their need to attend hospital appointments, so completing an anti-VEGF treatment regime can be a significant additional burden. Patients receiving intravitreal injections report high levels of anxiety and significant psychological impact [22]. The dependence on a caregiver to accompany the patient and the financial cost of treatment also pose a burden on the patient [23, 24].

This rapid review aims to identify and summarise the most recent information on the effectiveness of interventions to increase uptake and completion of treatment for DR and/or DMO in LMICs.

## Methods

### Rapid review question

What interventions are effective in increasing uptake and completion of treatment for DR and/or DMO among people with diabetes in LMICs?

### Protocol and registration

We used the STARR Decision Tool and the WHO practical guide to carefully consider the methods most appropriate for this rapid review and the possible limitations introduced by those methods [25, 26].

Additional file 1 provides the adapted Preferred Reporting Items for Systematic Reviews and Meta-analysis Protocols (PRISMA-P) which is used to report this protocol.

The protocol was registered on the Open Science Framework (OSF): osf.io/h5wgr; more details are provided in Additional file 2. Any protocol amendments will be documented in the registration site [27].

### Eligibility criteria

We will limit the search to peer-reviewed publications of studies conducted in LMICs in any setting in the last 20 years (i.e. community or facility based), which examine the uptake and/or completion of treatment prescribed for DR or DMO. We defined LMICs according to the World Bank Classification for 2019. Studies will only be included if they report at least one of the first four outcomes. We will restrict to publications in the English language. We will only include studies where a full-text report is available (Table 1).

**Table 1:** Eligibility criteria

Population	Adults (aged ≥ 18 years) with type 1 or type 2 diabetes in LMICs who have been identified as needing treatment for DR and/or DMO
Intervention	Any intervention that seeks to increase uptake and/or completion of treatment for DR and/or DMO among adults with diabetes
Comparator	Usual care or another intervention seeking to increase uptake and/or completion of treatment for DR and/or DMO
Outcomes	1. Proportion of patients initiating treatment for DR and/or DMO among those to whom it is recommended 2. Proportion of patients completing treatment for DR and/or DMO among those to whom it is recommended 3. Proportion of patients completing treatment for DR and/or DMO among those initiating treatment 4. Number and proportion of DR and/or DMO rounds of treatment completed per patient, as dictated by the treatment protocol 5. Cost-effectiveness of intervention to increase DR/DMO treatment uptake or completion
Study design	Any interventional or observational comparative study

### Information sources

We will search MEDLINE, Embase, Global Health and the Cochrane Register of Studies. The search strategies will be developed by a Cochrane Eyes and Vision Information Specialist (IG). We will search the reference lists of included studies. Grey literature will not be considered.

### Search strategy

The search strategy is included in Additional file 3.

### Data management and selection process

Screening will be done using online review management software (Covidence, Veritas Health Innovation, Melbourne, Australia; available at www.covidence.org). Each title and abstract will be screened independently by two reviewers. Disagreements will be resolved by discussion. Full-text articles describing potentially relevant studies will be screened by two reviewers independently against the inclusion and exclusion criteria and any differences resolved by consensus. A PRISMA flow diagram will be completed to summarise the study selection process.

### Risk of bias assessment

Risk of bias will be assessed by two reviewers independently using SIGN critical appraisal checklists for this purpose, available at https://www.sign.ac.uk. Any differences will be agreed by consensus, and a third researcher will be consulted if any differences are unresolved. The overall risk of bias for included studies will be reported in narrative form and used to interpret the findings of the review.

### Data extraction

A custom-designed Excel data extraction form will be piloted by two reviewers on 3 papers and adapted accordingly. Data will be extracted by one reviewer, and the accuracy of data extraction will be checked by a second reviewer for 10% of studies. If there are significant data extraction errors (for example, important errors in more than 1% of records), then a further set of records will be checked. The following data items will be collected for each identified intervention (Table 2).

**Table 2:** Data items

Data categories	Example data outcomes
Publication characteristics	Authors, title, publication date, journal citation
Study characteristics	Design
	Dates of data collection
Population characteristics	Country
	Population demographics (age, gender)
	Sample size
Intervention characteristics	Setting (rural/urban, community/hospital)
	Intervention target: patients, providers, policy-makers
	Treatment indication: pan retinal laser photocoagulation (PRP) for PDR, focal laser, intravitreal anti-VEGF or steroids for DMO
	Treatment completion guideline, e.g. number of laser sessions, number of burns and number of injections
	Type of intervention, e.g. guidelines, treatment regime, educational programmes, financial subsidies, follow-up prompts and reminders, ongoing quality assurance and improvement processes, electronic patient management
	Factors associated with uptake/completion of treatment: provider and patient
Outcomes	Proportion of patients initiating treatment for DR and/or DMO among those to whom it is recommended
	Proportion of patients completing treatment for DR and/or DMO among those to whom it is recommended
	Proportion of patients completing treatment for DR and/or DMO among those initiating treatment
	Number and proportion of DR and/or DMO rounds from the recommended treatment protocol completed per patient
	Cost-effectiveness of intervention to increase DR/DMO treatment uptake or completion

### Data synthesis

We anticipate that there will be clinical and methodological diversity in the studies that we find, so we plan to summarise the data narratively, following SWiM reporting guidance: Synthesis Without Meta-analysis [28].

We will report outcomes in terms of proportions in intervention and comparator groups and will calculate the risk ratio with 95% confidence intervals as the main measure of effect, but this will be dependent on how the data are reported by the included studies. In observational studies, for example, adjusted odds ratios may be the most appropriate measure of effect. We will present key characteristics such as study design, sample size and risk of bias in tables and use visual displays for effect estimates when possible. We will consider heterogeneity by examining study design, geographical location, demographic characteristics such as age and sex as well as the nature of the interventions and the settings in which they have been applied. If sufficient data are available, we will calculate *I*^2^ which is a measure of the percentage of variation across studies that is due to heterogeneity rather than chance. We will use the GRADE approach, as applied to narrative syntheses, to assess the certainty of the synthesis findings [29].

Depending on the findings, we will group studies by setting, intervention target (patients, health providers, health system), type of intervention, study design and outcomes. For each comparison and outcome, we will provide a description of the findings alongside the certainty of the evidence, ensuring consistency with the review question and providing a judgement as to the extent to which the studies contribute to the synthesis.

## Discussion

This rapid review aims to identify and synthesise the peer-reviewed literature on the effectiveness of interventions to increase uptake and completion of treatment for DR and/or DMO in LMICs. The rapid review methodology was chosen in order to rapidly synthesise the available evidence to support programme implementers and policy-makers in designing evidence-based health programmes and public health policy and inform the allocation of resources. This review may also identify gaps in the evidence that could inform further research priorities related to the management of diabetic retinopathy in LMICs.

## Supplementary Information

**Additional file 1:.** Reporting standards – PRISMA-P Checklist.

**Additional file 2:.** Registration OSF: https://osf.io/h5wgr/

**Additional file 3:.** Search strategy.

## Data Availability

Not applicable—no data sets currently available.

## Abbreviations

DR: Diabetic retinopathy
DMO: Diabetic macular oedema
LMIC: Low- and middle-income country
VTDR: Vision-threatening diabetic retinopathy
OSF: Open science framework
PDR: Proliferative diabetic retinopathy
VEGH: Vascular endothelial growth factor
ARMD: Age-related macular degeneration
PRISMA-P: Preferred reporting items for systematic reviews and meta-analysis protocols
SING: Scottish intercollegiate guidelines network
PRP: Pan retinal laser photocoagulation
SWiM: Synthesis without meta-analysis
GRADE: Grading of recommendations assessment, development and evaluation
