# Supplementary material for: Effectiveness of interventions to increase uptake and completion of treatment for diabetic retinopathy in low- and middle-income countries: a rapid review protocol
Source: Syst Rev. 2021 Jan 14;10:27. doi: 10.1186/s13643-020-01562-9 (PMC7809874; doi:10.1186/s13643-020-01562-9)
Supplement: Supplementary file 2 — Additional file 2:. Registration OSF: https://osf.io/h5wgr/ [file 13643_2020_1562_MOESM2_ESM.docx]

**Additional File 2: Protocol Registration details**

This protocol was registered on OSF on 29-04-2020

Citation:

Bascaran, C., Mwangi, N., D’Esposito, F., Cleland, C. R., Ulloa, J. A. L., Gordon, I., … Burton, M. (2020, October 2). Effectiveness of interventions to increase uptake and completion of treatment for diabetic retinopathy in low- and middle-income countries: a rapid review protocol. Retrieved from osf.io/h5wgr
