# Supplementary material for: Effectiveness of interventions to increase uptake and completion of treatment for diabetic retinopathy in low- and middle-income countries: a rapid review protocol
Source: Syst Rev. 2021 Jan 14;10:27. doi: 10.1186/s13643-020-01562-9 (PMC7809874; doi:10.1186/s13643-020-01562-9)
Supplement: Supplementary file 3 — Additional file 3:. Search strategy. [file 13643_2020_1562_MOESM3_ESM.docx]

**Additional file 2: search strategy**

**Effectiveness of interventions to increase uptake and completion of treatment for diabetic retinopathy in low- and middle-income countries: a rapid review protocol.**

Covadonga Bascaran^1^, Nyawira Mwangi^1,2^, Charles Cleland^1^, Fabrizio D’Esposito^3^, Iris Gordon^1^, Juan Alberto Lopez Ulloa^4^, Ranad Maswadi^5^, Shafi Mdala^6^, Jacqueline Ramke^1,7^, Jennifer R Evans^1^, Matthew Burton^1,8^

Affiliation

^1^London School of Hygiene and tropical Medicine, London, United Kingdom

^2^Kenya Medical Training College, Nairobi, Kenya

^3^ The Fred Hollows Foundation, Melbourne, Australia

^4^ Instituto Nacional de Oftalmologia, Queretaro, Mexico

^5^ St Thomas’ Hospital, London

^6^ Queen Elizabeth Central Hospital, Blantyre, Malawi

^7^ School of Optometry and Vision Science, University of Auckland, Auckland, New Zealand

^8^ Moorfields Eye Hospital, London, United Kingdom

**Medline Ovid search strategy**

1. Diabetic Retinopathy/

2. ((diabet$ or proliferative or non-proliferative) adj4 retinopath$).tw.

3. diabetic retinopathy.kw.

4. (diabet$ adj3 (eye$ or vision or visual$ or sight$)).tw.

5. (retinopath$ adj3 (eye$ or vision or visual$ or sight$)).tw.

6. (DR adj3 (eye$ or vision or visual$ or sight$)).tw.

7. or/1-6

8. Macular Edema/

9. (macula$ adj3 oedema).tw.

10. (macula$ adj3 edema).tw.

11. maculopath$.tw.

12. (CME or CSME or CMO or CSMO).tw.

13. (DMO or DME).tw.

14. or/8-13

15. exp diabetes mellitus/

16. diabetes complications/

17. diabet$.tw.

18. or/15-17

19. 14 and 18

20. 7 or 19

21. exp Patient Acceptance of Health Care/

22. exp Decision Making/

23. (adhere or adherence or compliance or compliant or noncomplian$ or non-complian$ or uptake or takeup).tw.

24. ((accept$ or initiat$ or default$) adj3 (treat$ or regimen)).tw.

25. ((patient$ or participant$ or person or people or individual$) adj5 (complet$ or follow$) adj3 (treat$ or regimen)).tw.

26. (health adj2 seek$ adj2 behavio$).tw.

27. (fear adj3 (treat$ or laser$ or inject$)).tw.

28. (anxi$ adj3 (treat$ or laser$ or inject$)).tw.

29. "do not present".tw.

30. (no adj1 show).tw.

31. (reminder adj2 (appointment$ or reappointment$)).tw.

32. (travel$ or chaperone).tw.

33. or/21-32

34. exp lasers/

35. laser$.tw.

36. exp light coagulation/

37. photocoagulat$.tw.

38. (photo adj1 coagulat$).tw.

39. (coagulat$ or argon or krypton or YAG or diode or micropulse or Pascal or panretinal).tw.

40. exp antibodies, monoclonal, humanized/

41. angiogenesis inhibitors/

42. angiogenesis inducing agents/

43. endothelial growth factors/

44. exp vascular endothelial growth factors/

45. (anti adj2 VEGF$).tw.

46. (endothelial adj2 growth adj2 factor$).tw.

47. (anti adj1 angiogen$).tw.

48. (macugen$ or pegaptanib$ or lucentis$ or rhufab$ or ranibizumab$ or bevacizumab$ or avastin or aflibercept$ or conbercept$ or OPT 302 or Opthea$ or RTH258 or Brolucizumab$ or abicipar pegol).tw.

49. VEGF TRAP$.tw.

50. exp steroids/

51. triamcin$.tw.

52. dexamethasone$.tw.

53. fluocinolone.tw.

54. (steroid$ or glucocorticoid$).tw.

55. or/34-54

56. 20 and 33 and 55

57. exp case reports/

58. (case adj1 (report$ or stud$)).tw.

59. 57 or 58

60. 56 not 59

61. (animal or mice or mouse or rat or rats or cell$ or inflammation or binding or aptamer).ti.

62. 60 not 61

63. limit 62 to english language

64. limit 63 to yr="2000 -Current"

**Embase OVID search strategy**

1. exp diabetic retinopathy/

2. ((diabet$ or proliferative or non-proliferative) adj4 retinopath$).tw.

3. diabetic retinopathy.kw.

4. (diabet$ adj3 (eye$ or vision or visual$ or sight$)).tw.

5. (retinopath$ adj3 (eye$ or vision or visual$ or sight$)).tw.

6. (DR adj3 (eye$ or vision or visual$ or sight$)).tw.

7. or/1-6

8. diabetic macular edema/

9. (diabe$ adj3 macula$ adj3 oedema).tw.

10. (diabe$ adj3 macula$ adj3 edema).tw.

11. (DMO or DME).tw.

12. or/8-11

13. 7 or 12

14. patient attendance/ or patient compliance/ or patient dropout/ or patient participation/ or patient preference/ or refusal to participate/ or treatment interruption/ or treatment refusal/

15. patient decision making/

16. (adhere or adherence or compliance or compliant or noncomplian$ or non-complian$ or uptake or takeup).tw.

17. ((accept$ or initiat$ or default$) adj3 (treat$ or regimen)).tw.

18. ((patient$ or participant$ or person or people or individual$) adj5 (complet$ or follow$) adj3 (treat$ or regimen)).tw.

19. (health adj2 seek$ adj2 behavio$).tw.

20. (fear adj3 (treat$ or laser$ or inject$)).tw.

21. (anxi$ adj3 (treat$ or laser$ or inject$)).tw.

22. "do not present".tw.

23. (no adj1 show).tw.

24. (reminder adj2 (appointment$ or reappointment$)).tw.

25. (travel$ or chaperone).tw.

26. or/14-25

27. exp laser coagulation/

28. argon laser/

29. laser$.tw.

30. photocoagulat$.tw.

31. (photo adj1 coagulat$).tw.

32. (coagulat$ or argon or krypton or YAG or diode or micropulse or Pascal or panretinal).tw.

33. exp angiogenesis inhibitors/

34. angiogenesis/

35. angiogenic factor/

36. endothelial cell growth factor/

37. monoclonal antibody/

38. vasculotropin/

39. (anti adj2 VEGF$).tw.

40. (endothelial adj2 growth adj2 factor$).tw.

41. (anti adj1 angiogen$).tw.

42. (macugen$ or pegaptanib$ or lucentis$ or rhufab$ or ranibizumab$ or bevacizumab$ or avastin or aflibercept$ or conbercept$ or OPT 302 or Opthea$ or RTH258 or brolucizumab$ or abicipar pegol).tw.

43. exp steroid/

44. triamcin$.tw.

45. dexamethasone$.tw.

46. fluocinolone.tw.

47. (steroid$ or glucocorticoid$).tw.

48. or/27-47

49. 13 and 26 and 48

50. exp case report/

51. (case adj1 (report$ or stud$)).tw.

52. 50 or 51

53. 49 not 52

54. (animal or mice or mouse or rat or rats or cell$ or inflammation or binding or aptamer).ti.

55. 53 not 54

56. limit 55 to conference abstract status

57. 55 not 56

58. limit 57 to english language

59. limit 58 to yr="2000 -Current"

**Global Health OVID search strategy**

1. ((diabet$ or proliferative or non-proliferative) adj4 retinopath$).tw.

2. (diabet$ adj3 (eye$ or vision or visual$ or sight$)).tw.

3. (retinopath$ adj3 (eye$ or vision or visual$ or sight$)).tw.

4. (DR adj3 (eye$ or vision or visual$ or sight$)).tw.

5. (diabe$ adj3 macula$ adj3 oedema).tw.

6. (diabe$ adj3 macula$ adj3 edema).tw.

7. (DMO or DME).tw.

8. or/1-7

9. patient compliance/

10. (adhere or adherence or compliance or compliant or noncomplian$ or non-complian$ or uptake or takeup).tw.

11. ((accept$ or initiat$ or default$) adj3 (treat$ or regimen)).tw.

12. ((patient$ or participant$ or person or people or individual$) adj5 (complet$ or follow$) adj3 (treat$ or regimen)).tw.

13. (health adj2 seek$ adj2 behavio$).tw.

14. (fear adj3 (treat$ or laser$ or inject$)).tw.

15. (anxi$ adj3 (treat$ or laser$ or inject$)).tw.

16. "do not present".tw.

17. (no adj1 show).tw.

18. (reminder adj2 (appointment$ or reappointment$)).tw.

19. (travel$ or chaperone).tw.

20. or/9-19

21. lasers/

22. laser$.tw.

23. photocoagulat$.tw.

24. (photo adj1 coagulat$).tw.

25. ((focal or grid) adj3 laser$).tw.

26. (coagulat$ or argon or krypton or YAG or diode or micropulse or Pascal or panretinal).tw.

27. exp monoclonal antibodies/

28. (anti adj2 VEGF$).tw.

29. (endothelial adj2 growth adj2 factor$).tw.

30. (anti adj1 angiogen$).tw.

31. (macugen$ or pegaptanib$ or lucentis$ or rhufab$ or ranibizumab$ or bevacizumab$ or avastin or aflibercept$ or conbercept$ or OPT 302 or Opthea$ or RTH258 or Brolucizumab$ or abicipar pegol).tw.

32. VEGF TRAP$.tw.

33. exp steroids/

34. triamcin$.tw.

35. dexamethasone$.tw.

36. fluocinolone.tw.

37. (steroid$ or glucocorticoid$).tw.

38. or/21-37

39. 8 and 20 and 38

40. exp case reports/

41. (case adj1 (report$ or stud$)).tw.

42. 40 or 41

43. 39 not 42

44. (animal or mice or mouse or rat or rats or cell$ or inflammation or binding or aptamer).ti.

45. 43 not 44

46. limit 45 to english language

47. limit 46 to yr="2000 -Current"

Cochrane Register of Studies search strategy

#1 MeSH descriptor: [Diabetic Retinopathy] this term only

#2 (diabet* or proliferative or non-proliferative) NEAR/4 retinopath*

#3 diabet* NEAR/3 (eye* or vision or visual* or sight*)

#4 retinopath* NEAR/3 (eye* or vision or visual* or sight*)

#5 DR NEAR/3 (eye* or vision or visual* or sight*)

#6 #1 OR #2 OR #3 OR #4 OR #5

#7 MeSH descriptor: [Macular Edema] this term only

#8 macula* NEAR/3 oedema

#9 macula* NEAR/3 edema

#10 maculopath*

#11 CME or CSME or CMO or CSMO

#12 DMO or DME

#13 #7 OR #8 OR #9 OR #10 OR #11 OR #12

#14 MeSH descriptor: [Diabetes Mellitus] explode all trees

#15 MeSH descriptor: [Diabetes Complications] explode all trees

#16 diabet*

#17 #14 OR #15 OR #16

#18 #13 AND #17

#19 #6 OR #18

#20 MeSH descriptor: [Patient Acceptance of Health Care] explode all trees

#21 MeSH descriptor: [Decision Making] explode all trees

#22 adhere or adherence or compliance or compliant or noncomplian* or non-complian* or uptake or takeup

#23 (accept* or initiat* or default*) near/3 (treat* or regimen)

#24 (patient* or participant* or person or people or individual*) near/5 (complet* or follow*) near/3 (treat* or regimen)

#25 health near/2 seek* near/2 behavio*

#26 fear near/3 (treat* or laser* or inject*)

#27 anxi* near/3 (treat* or laser* or inject*)

#28 "do not present"

#29 no near/1 show

#30 reminder near/2 (appointment* or reappointment*)

#31 travel* or chaperone

#32 #20 OR #21 OR #22 OR #23 OR #24 OR #25 OR #26 OR #27 OR #28 OR #29 OR #30 OR #31

#33 MeSH descriptor: [Lasers] explode all trees

#34 laser*

#35 MeSH descriptor: [Light Coagulation] explode all trees

#36 photocoagulat*

#37 photo NEXT coagulat*

#38 coagulat* or argon or krypton or YAG or diode or micropulse or Pascal or panretinal

#39 MeSH descriptor: [Antibodies, Monoclonal, Humanized] explode all trees

#40 MeSH descriptor: [Angiogenesis Inhibitors] this term only

#41 MeSH descriptor: [Angiogenesis Inducing Agents] this term only

#42 MeSH descriptor: [Endothelial Growth Factors] this term only

#43 MeSH descriptor: [Vascular Endothelial Growth Factors] explode all trees

#44 anti near/2 VEGF*

#45 endothelial near/2 growth near/2 factor*

#46 anti NEXT angiogen*

#47 macugen* or pegaptanib* or lucentis* or rhufab* or ranibizumab* or bevacizumab* or avastin or aflibercept* or conbercept* or OPT 302 or Opthea* or RTH258 or brolucizumab* or abicipar pegol

#48 VEGF TRAP*

#49 MeSH descriptor: [Steroids] explode all trees

#50 triamcin*

#51 dexamethasone*

#52 fluocinolone*

#53 steroid* or glucocorticoid*

#54 #33 or #34 or #35 or #36 or #37 or #38 or #39 or #40 or #41 or #42 or #43 or #44 or #45 or #46 or #47 or #48 or #49 or #50 or #51 or #52 or #53

#55 #19 and #32 and #54 with Publication Year from 2000 to 2020, in Trials
